# Supplementary material for: Socioeconomic risk factors for fatal opioid overdoses in the United States: Findings from the Mortality Disparities in American Communities Study (MDAC)
Source: PLoS One. 2020 Jan 17;15(1):e0227966. doi: 10.1371/journal.pone.0227966 (PMC6968850; doi:10.1371/journal.pone.0227966)
Supplement: Attachment — Submitted filename: PONE-D-19-16947 Response to reviewers 9 30 19.docx [file pone.0227966.s002.docx]

**Response to Reviewers’ comments: PONE-D-19-16947**

S Altekruse, C Cosgrove, W Altekruse, R Jenkins, C Blanco. Socioeconomic risk factors for fatal opioid overdoses in the United States: Findings from the Mortality Disparities in American Communities Study

**Journal Editorial Department**

**Journal Editorial Comment 1:** Please clarify all data that constitutes minimal data and where it is publicly available (e.g. manuscript Results section, Supporting Information files, public stable repository)

**Response:** On lines 101-105, all constituent variables of the minimal dataset for the analysis are listed:

*“The MDAC reference manual [12] provides detailed description of underlying variables in the present analysis. The minimal data for analysis was comprised of the following variables: cause of death, age, race, Hispanic ethnicity, sex, disability, marital status, employment status, educational attainment, citizenship, housing tenure, rurality, health insurance status, incarceration, household poverty, and Census Division.”*

On lines 149-153, processes that investigators can use to access the data are described:

*“**In compliance with privacy protection requirements of U.S. Code Title 13, data access was obtained by submitting a research proposal form to the MDAC Steering Committee. We accessed data with Census analyst assistance rather than at a Research Data Center, two processes available to all investigators. [12] Output was reviewed by the Census Bureau Disclosure Review Board to ensure confidentiality.”*

**Journal Editorial Comment 2:** Please fully disclose the methods of data collection (perhaps MDAC reference manual in Methods section) and confirm the authors of the present study had no special access privileges in accessing MDAC which other interested researchers would not have.

**Response:** On lines 84-89, the methods of data collection are described:

*“The 2008 wave of the ACS [13] was linked to NDI [14] death certificate records from 2008 to 2015 to create the nationally representative MDAC database of children and adults. Linkage was based on either social security number or the dyad of first and last name plus date of birth. Up to 10 possible matches per ACS record were returned to the Census Bureau. Results were then run through a Census Bureau algorithm that used address, family member names, and other demographic and SES characteristics to assess whether a match was a true match.”*

On lines 151-152 we indicate that the authors no special access privileges in accessing MDAC:

*“We accessed data with Census analyst assistance rather than at a Research Data Center, two processes available to all investigators. [12] In compliance with privacy protection requirements of U.S. Code Title 13, data access was obtained by submitting a research proposal form to the MDAC Steering Committee. [12] Data were accessed with Census Bureau analyst assistance rather than at a Research Data Center, the two processes available to all investigators to access the full MDAC dataset. ”*

In the **Access to Data** Statement, on lines 355-359 the authors reconfirm that they had no special privilege in accessing the MDAC dataset that other interested researchers would not have, before again describing the mechanism of data access:

*“The authors of the present study had no special access privileges in accessing MDAC which other interested researchers would not have.* *In compliance with privacy protection requirements of U.S. Code Title 13, data access was obtained by submitting a research proposal form to the MDAC Steering Committee. [12] Data were accessed with Census Bureau analyst assistance rather than at a Research Data Center, the two processes available to all investigators to access the full MDAC dataset.”*

**Academic Editor**

**Academic Editor Comment 1:** What was the rationale/approach for variable selection in the adjusted analysis?

**Response:** We thank the editor for this suggestion, which greatly improved the quality of the analysis. On page 7, line 131-134 the method for selecting referent groups is provided.

*“**Referent groups were selected based on a priori information on groups with low risk of opioid overdose death, with consideration to ensure sufficient numbers of events occurred in the referent group to estimate stable hazard ratios. Thus Hispanics were the selected referent race/ethnicity group and continuous age and age squared variables (to adjust for nonparametric distribution) were used to analyze the effect of age.”*

On page 7, line 135-139, the rationale for presenting a partially adjusted model with key demographic variables and a single SES variable are described.

*“In addition to univariate models, partially adjusted models included key demographic attributes (age, sex, race/ethnicity) and a single SES or geographic variable. The final model included demographic and SES attributes retained after forward stepwise model selection. This method enabled evaluation of changes in effects as multiple demographic and SES covariates of interest were added to proportional hazard models. [16]”*

On page 7, line 139-141, the approach to retention or elimination of variables when known collinearity exists is described.

*“To limit effects of collinear variables, educational attainment and employment status were retained while occupation was eliminated from analysis. Similarly, citizenship status was retained while place of birth was eliminated.”*

The forward stepwise model selection process used in the final model is described on page 7, lines 141-149.

*“For the purpose of final model development, we started with no predictor variables and used a forward stepwise regression process (PROC PHREG, SAS v9.4) to select one variable at a time based on the statistical significance of the Chi-squared test of the regression model parameter estimates. All 15 candidate variables of interest were retained in the final model, listed here in the order of their stepwise forward selection into the model: disability status, marital status, employment status, age squared (to adjust for nonparametric distribution), continuous age, race/ethnicity, sex, educational attainment, [17] citizenship, employment status, housing status, rural versus nonrural residence, [18] health insurance status, incarceration status at time of survey, household poverty level, and location of residence across nine Census Bureau Divisions of the United States [19].”*

**Academic Editor Comment 2:** There is no rationale provided for base, base+one SES factor and final models.

**Response:** On page 7, line 135-139, rationale is provided for univariate models, partially adjusted models included key demographic attributes (age, sex, race/ethnicity) and a single SES or geographic variable.

*“In addition to univariate models, partially adjusted models included key demographic attributes (age, sex, race/ethnicity) and a single SES or geographic variable, as well as a final model that included all demographic as well as SES attributes. This enabled evaluation of effects as multiple demographic and SES covariates of interest were added to proportional hazard models. [16]”*

**Academic Editor Comment 3:** There is no rationale for parameterization of factors nor selection of reference categories, leading to strange results of limited utility (i.e., 80+ for the reference group for age).

**Response:** In response to this helpful comment, referent groups for age and race/ethnicity were changed in the revised manuscript. On page 7, line 133-136, we describe changing the age variable from an age group category variable to a continuous variable with an age squared term to address potential non-parametric distribution of age and eliminate the small count 80+ group as a referent group. Similarly, with respect to race/ethnicity, the referent group was changed from Asian and Pacific Islander to Hispanic, providing more stable counts for Cox Proportional Hazard Analyses. This suggested revision improve the performance of final models with respect to these variables.

*“Referent groups were selected based on a priori information on groups with low risk of opioid overdose death, with consideration to ensure sufficient numbers of events occurred in the referent group to estimate stable hazard ratios. Thus Hispanics were the selected referent race/ethnicity group and continuous age and age squared variables (to adjust for nonparametric distribution) were used to analyze the effect of age.”*

**Academic Editor Comment 4:** Did you consider any effect modification analysis, for example to examine the relationship between covariates of interest (i.e., SES) and geographic region?

**Response:** We did not consider interaction terms in this analysis. The goals of this analysis were broad in nature. On page 27, line 347-348, as a limitation we note that many other MDAC analyses of opioid-related mortality are of interest.

*“In addition, to focus our analyses, we limited our analyses to SES-related variables. Future studies should build on our findings to examine additional predictors of opioid overdose death.”*

**Academic Editor Comment 5:** Given the national data, there could be further exploration of the data and the relationships examined in the paper.

**Response:** As indicated in response to the Editor’s comment 4 above we agree with this suggestion and note that this was an overview paper. Our goal is to make the MDAC database known and available as a resource to explore opioid fatality topics in depth in future papers.

**Academic Editor Comment 6:** Finally, there is limited information provided in the text regarding the measurement or meaning of the covariates included in the models.

**Response:** On line 101, readers are referred to the MDAC reference manual for detailed information on each variable used in the analysis:

*“The MDAC reference manual [12] describes all underlying variables used in this analysis. Minimal data for analysis was comprised of the following variables: cause of death, age, race, Hispanic ethnicity, sex, disability, marital status, employment status, educational attainment, citizenship, housing tenure, rurality, health insurance status, incarceration, household poverty, and Census Division.”*

In response to this comment, on page 5, starting on line 106, the ICD-10 variable for opioid deaths is more fully described.

*“International Classification of Diseases, 10th Revision (ICD–10) [15] mortality codes defined cause of death. Since non-specific fatal opioid overdoses account for the majority of fatal opioid death certificates [7], opioid overdoses were defined as a single category, using ICD–10 multiple-cause-of death codes T40.0-T40.4 and T40.6 (opium, heroin, other opioids, methadone, synthetic narcotics other than methadone, and unspecified narcotic; respectively).”*

The household poverty variable is now described in detail starting on line 110.

*“Poverty status was defined using the Census Bureau standard method, based on total family income in the past 12 months, family size and age composition. If total income of the householder's family was less than the 2008 threshold appropriate for that family, then the person was considered “below the poverty level,” together with every member of his or her family. The matrix tables for poverty level calculations do not vary across the 50 states and the District of Columbia.”*

Construction of age variables is described on line 133.

*“…continuous age and age squared variables (to adjust for nonparametric distribution) were used to analyze the effect of age.”*

Rural versus non-rural residence is described with reference on line 147. It is clarified that incarceration status was measured at time of survey on line 148. A reference to Census Divisions is provided on line 149.

**Reviewer 1**

**Reviewer 1 Comment 1:** Grounding the research in the large body of existing literature on fatal overdose would strengthen the paper.

**Response:** Thank you for this suggestion. The manuscript refence section was expanded by 10 references, primarily on this topic, including the excellent AJPH review articles by Dasgupta et al on socioeconomic status and the opioid epidemic and Martins et al on socioeconomic status of unintentional drug overdose that you recommended.

**Abstract**

**Reviewer 1 Comment 2:** The results section of the abstract should be revised; there appears to be incomplete reporting e.g., urban group had higher risk than the rural group but was not reported, “HRs near two” etc. and the wording is convoluted at times.

**Response:** We thank the reviewer for the suggestion, incorporated throughout the revised manuscript.

**Reviewer 1 Comment 3:** The abstract refers to “final models” when the results only report one final model.

**Response:** This typographical error was corrected.

**Reviewer 1 Comment 4:** I suggest rewriting the results section of the abstract to be more concise and more informative.

**Response:** This suggestion was adopted with careful attention to convey the most salient information.

**Introduction**

**Reviewer 1 Comment 5:** Page 3: Nationwide, fatal opioid overdoses have disproportionately [2] but not exclusively [3] affected men, and particularly adolescent, young and middle-aged adult white men. -Clarify whether this is referring to fatal opioid overdose prevalence or rates over time and the historical period of relevance.

**Response:** On line 63, it is now stated that, “Nationwide, the rising rate of fatal opioid overdoses has disproportionately affected…”

**Reviewer 1 Comment 6:** It would be worth mentioning the reason why overdose has been conceptualized as a death of despair to strengthen the rationale for focusing on SES, and to cite the original paper that developed the theory. The cited reference does not use the phrase and so the intended connection is unclear.

**Response:** On line 61, it is now stated, “This term provides a useful contextual framework for studying SES-related risk factors for fatal opioid overdose and interventions to treat and prevent them.” As recommended by the reviewer, we now cite reference 4, Case and Deaton’s original publication using the term “death of Despair.”

**Reviewer 1 Comment 7:** Given that this is a national study, more could be provided in the introduction about the relationship between overdose rates and geography to better contextualize the findings (e.g., urban vs. rural settings), including whether overdoses or low SES are known to be concentrated in certain locales.

**Response:** Continuing on lines 64-65, the introduction includes the following statement with references,

*“Highest opioid death rates are reported in Mountain, Rust Belt, New England states and the South. [7] In 2017, a shift was seen in urbanicity of the opioid epidemic, with higher reported death rates in urban than rural areas. [8] At-risk socioeconomic groups for fatal drug use include people in lower income strata, who did not graduate from high school, the homeless, and recently released prisoners. [9]”*

**Reviewer 1 Comment 8:** Much has been written about the current opioid epidemic. A description of the established risk factors of fatal overdose or at minimum including citations of published reviews (e.g., Dasgupta et al., AJPH; Martins et al., AJPH) would nicely set up the subsequent sections and lay the foundation for the known confounders (measured and unmeasured) that should be considered.

**Response:** As indicated in response to Reviewer 1, Comment 1 above, the manuscript refence section was expanded by 10 references, primarily on the topic of SES, including the recommended seminal AJPH review articles by Dasgupta et al on socioeconomic status and the opioid epidemic and Martins et al on socioeconomic status of unintentional drug overdose.

**Methods:**

**Reviewer 1 Comment 9:** Page 4: (12.8%) ACS respondents who were missing information necessary to meet the NCHS minimum criteria required for matching – did the authors conduct secondary analyses to understand the degree of bias due to this missingness e.g., if this group differed on socio-demographics?

**Response:**  On line 120-123, we state, “*Census Bureau evaluation of the age, race/ethnicity, and gender of MDAC respondents who were included in MDAC and those without sufficient information to complete an ACS/NDI linkage revealed no biases with respect to these variables [personal communication, Norman Johnson].”* ”

**Reviewer 1 Comment 10:** Page 5: The Census Bureau definition of rural [14] excluded metropolitan and micropolitan areas, incorporated census-designated places with populations of 2500 or more. Residents were classified as rural or urban. It is an important factor with implications for targeting resources. Rural vs. nonrural is a more accurate label. Could the authors use more granular classification?

**Response:** We thank the reviewer for this comment. On line 147, the variable is renamed “rural vs. nonrural.” A reference defining the variable is provided. On line 289 we indicate that people residing in nonrural areas accounted for 79% of opioid deaths while rural residents accounted for 21% of opioid deaths. This finding is discussed in the context of the literature in the paragraph beginning on line 289. While this MDAC analysis does not include a more granular variable for residential density, this variable does appears relevant in the context of the recent literature showing a shift of the plurality of opioid fatalities away from rural settings.

**Reviewer 1 Comment 11:** Page 6: Analyses that accounted for multistage sampling in the ACS were conducted using the PHREG procedure – clarify the number of levels included in the final model. Did this method account for correlations between individuals surveyed from the same area of residence (e.g., census division)?

**Response:** The multistage variables used in sample weight assignment are described on line 90-93.

*“The complex sampling frame for the ACS is derived from the Master Address File. Sampling is designed to approximate characteristics observed in the Census Bureau’s annual United States population estimates for the following attributes:* *age, sex, race, Hispanic ethnicity, and state of residence. Analyses did not account for correlations between individuals within each level for these variables.”*

**Reviewer 1 Comment 12:** Page 6: incarceration at time of survey – clarify here and in the table whether this is referring to the setting in which the survey was conducted or if the individual had a history of incarceration.

**Response:** On line 148 and in tables it is stated that incarceration status was measured at time of survey.

**Reviewer 1 Comment 13:** Page 6: Were any confounders considered for inclusion in the regression analysis? Access to naloxone is an example. If not, this should be stated in the limitations.

**Response:** On line 343, it is stated that data on access to naloxone was not available.

**Results:**

**Reviewer 1 Comment 14:** Page 8: Many variables as modeled contain extremely small cell sizes (e.g., n=36 among ages 80+; n=29 among Asian Pacific Islanders; n=43 American Indian Alaskan Native etc), which calls into question the validity of these results. Where appropriate, I would suggest re-categorization/collapsing those categories into another category to generate larger cell sizes and/or replacing reference groups containing small cells with new reference groups with larger samples; this will affect the precision of the Cox regression models.

**Response:** We appreciate this suggestion, and referent groups for age and race/ethnicity were changed in the revised manuscript. Additional information is provided in response to Editor Comment 1.

**Reviewer 1 Comment 15:** Table 2: Clarify whether the presented data are from the Final adjusted model or the Partially adjusted model

**Response:** Thank you for pointing this out. On line 192 it is now stated, *“In the final model, results for fatal opioid overdose included the following:”*

**Discussion**

**Reviewer 1 Comment 16:** Page 22: This nationally representative descriptive study – given that the paper includes data analysis, it is observational rather than descriptive.

**Response:** In response to this helpful comment, line 235 was revised as follows:

*“The nationally representative MDAC observational study provides insights into longitudinal relationships between SES and opioid-related mortality.”*

**Reviewer 1 Comment 17:** Page 22: Given that the term “opioid” is often used synonymously with prescription opioid, I suggest highlighting in the opening paragraph that the analysis also included deaths involving non-medical opioids including heroin and fentanyl.

**Response:** Line 238 states opioids include medical opioids, heroin, fentanyl and its synthetic analogues.

**Reviewer 1 Comment 18:** Page 22: What do the authors mean by “rehabilitate those at-risk for opioid overdose?” Replacing this with less stigmatizing language is recommended.

**Response:** Line 244-245 has been revised to be less stigmatizing:

*“**These findings may be of use in developing more targeted efforts to prevent fatal opioid overdoses.”*

**Reviewer 1 Comment 19:** Page 22: were any subpopulations undersampled? E.g., Asian Pacific Islanders; American Indian Alaskan Native Americans. How valid are the findings around race/ethnicity?

**Response:** Line 95-97 was revised as follows: *“* *Whites were oversampled by about 2% compared to the 2010 Census, and therefore this group was assigned a weight of slightly less than one. Other racial/ethnic groups were undersampled by one percent or less and thus given weights above one.”*

**Reviewer 1 Comment 20:** Page 23: How does the poverty measure used account for regional cost of living differences?

**Response:** This is addressed on lines 110 through 115:

*“Derivation of the poverty status variable is described in detail because more than one method is in common use. The variable we used is the standard Census Bureau procedure, involving total family income in the past 12 months, family size and age composition. If the total income of the householder's family was less than the 2008 threshold appropriate for that family, then the person was considered “below the poverty level,” together with every member of his or her family. Matrix tables for poverty level calculations do not vary across the 50 states and D.C.”*

**Reviewer 1 Comment 21:** Page 24: Compared to people who owned a house with a mortgage, renters or owners without a mortgage were at risk of fatal opioid overdose during the study period – what was the hypothesis? Was this a surprising finding? I would suggest explaining this finding further.

**Response:** Line 286-289 addresses this helpful reviewer’s comment:

*“An unexpected finding in the present study was that homeowners with mortgages were at elevated risk of opioid death compared to those with a mortgage. A plausible explanation is that the pressure to make scheduled mortgage payments could impose a routine structure into daily life that discourages opioid misuse.”*

**Reviewer 1 Comment 22:** Page 24: The availability and lethality of fentanyl – I suggest clarifying that it is mostly illicitly manufactured fentanyl.

**Response:** This suggestion is also appreciated. Beginning on line 327 the following revision was made:

*“**These later onset post-incarceration deaths were not thought to be from primarily overdoses due to reduced tolerance but the lethality of mostly illicit fentanyl when encountered.”*

**Reviewer 1 Comment 23:** Page 24: Increased access to opioid agonist therapy for treating opioid use disorder may have merit, but currently adherence rates are low – it is unclear what is meant by “low” and what the possible underlying reasons may be. This statement does not add to the discussion and could be removed.

**Response:** The statement beginning on line 327 is replaced with,

*“**Ongoing efforts to improve transition of care for people with psychiatric disorders in the months around leaving prison could help to decrease the number of overdoses.”*

**Reviewer 1 Comment 24:** Limitations: How stable are the SES indicators over time? It would be helpful if the authors could explain which SES variables are most susceptible to temporal change and which remain relatively stable over time.

**Response:** It would be difficult to rank the SES variables used in this analysis based on the probability of change overtime. On line 344, we attempt to address this limitation as follows:

*“Furthermore, time-varying measures that can change over time (e.g., employment, health insurance) were assessed once, up to 7 years before death.”*

**Reviewer 1 Comment 25:** Limitations: Homelessness is highly prevalent among people who misuse opioids, and many of those who overdose are unstably housed.

**Response:** The ACS does not ask about the stability of housing. Beginning on line 284, we discuss how the ACS data on housing tenure is consistent with other data on drug use by housing stability. We also cite lack of detail on housing stability as a limitation on line 345:

*“…we limited our analyses to SES-related variables, some of which lacked optimal detail (e.g., homelessness with housing tenure and incarceration status at time of death).”*

**Reviewer 1 Comment 26:** Same with individuals who are incarcerated at the time of the study. How does the ACS account for homeless and incarcerated individuals? If this is a limitation of the study, please state.

**Response:** In the methods, results and discussion we state that incarceration was determined at the time of the 2008 ACS survey. On line 346, limited detail on incarceration status is listed as a limitation.

**Reviewer 1 Comment 27:** Minor comments: typographic errors should be fixed throughout.

**Response:** We have proof read the manuscript in an effort to eliminate typographical errors.

**Reviewer #2:**

**Abstract:**
**Reviewer 2 Comment 1:** In the abstract, mentioning the American Community Survey (versus MDAC), is a bit confusing, since MDAC is mentioned in the title. I would suggest being consistent from the title to the abstract for those readers who are doing a brief read of your paper. Also, you could clarify that the SES attributes are based on the time of American Community Survey participation.
**Response:** Line 26-27 were revised as follows:

*“The Mortality Disparities in American Community Study (MDAC) links population-weighted 2008 American Community Survey responses (n=3,934,000) to the National Death Index through 2015.”*

**Introduction**
**Reviewer 2 Comment 2:** The introduction to this paper is very brief and the first paragraph is a bit disjointed, briefly covering opioid overdose mortality (the focus of this paper), other deaths of despair (which I’m not sure is needed), and SES disparities. In the second paragraph, the authors circle back to state that there is existing research on demographic and geographic trends in opioid overdoses. The introduction may better justify the analysis if it is expanded. For example, the authors could structure the introduction to present the existing research on opioid overdose mortality and disparities, highlighting the lack of socioeconomic detail. In the second paragraph, they could introduce and expand the existing information on socioeconomic disparities in life expectancy that may be driven by opioid overdoses and other deaths of despair. They could also further explain some of the existing research on distressed communities to motivate looking more into SES indicators.
**Response:** We appreciate this constructive comment. It was informative in our effort to revise the introduction. The number of citations in the paper was expanded to reference review articles on relationships between SES and drug overdose by Martins et al and Dasgupta et al. The work of Case and Deaton on deaths of despair was also cited, because it provides a framework for examining the relationship between SES indicators and fatal opioid overdoses.

**Methods**
**Reviewer 2 Comment 3:** What are the NCHS minimum criteria for matching to the NDI you mention in line 84 (i.e. same as those in line 93-94)? Could you add these or provide a reference? Also, define NCHS at first mention in line 84 (later you spell it out without specifying that this is NCHS is lines 92-93) and it makes it seem like there are multiple criteria/procedures.
**Response:** This sentence was revised and is now found on line 119:

*“The 578,000 (12.8%) ACS records without data for NDI linkage were dropped from the study (i.e., social security number or name and date of birth).”*

**Reviewer 2 Comment 4:** Please clarify what you mean by ‘centered on one’: “Frequencies were estimated using weighted data adjusted to be *centered on one*, with rounding to four significant digits to protect against disclosure of individual information.”

**Response:** Lines 97-99 are revised to indicate weights are centered on one observation per response:

*“Overall weights were centered on one observation per respondent, so that the weighted sample approximates the number of 2008 ACS responses rather than the U.S. population.’*

**Results**
**Reviewer 2 Comment 5:** Table 1 has a footnote that says the cell frequencies are based on weighted data, but the frequencies add to the number that I understood to be the unweighted/sampled n ~3.9 million.

**Response:** Please see the response to Reviewer 2, Comment 4.

**Reviewer 2 Comment 6:** The clarity of Table 2 might be improved by presenting the first column as the ‘Base Or Partially Adjusted Model’ – it is a bit confusing to only call that column the Base model and include HRs for SES covariates.
**Response:** We appreciate this suggestion. Table 2 now labels the three sets of models as the univariate models, partially adjusted models, and final model. The footnote for the partially adjusted model defines the variables in the models (i.e., age variables, non-Hispanic race/Hispanic ethnicity, and sex alone or with one SES or geographic variable).

**Reviewer 2 Comment 7:** The results section describing the HRs is quite long and needs proofreading. I would suggest condensing this section and only including the key findings to make sure they are not overlooked. For example, you begin the results section describing Table 2/regression models by saying “HRs for many age groups were higher in final than base models (Table 2).” I’m not sure the point of highlighting this since the interest is in the SES variables.

**Response:** The authors appreciate this comment and have incorporated it into the revised results section, by not emphasizing the effects for the age, a demographic rather than SES variable, in various models.

**Reviewer 2 Comment 8:** Please also be consistent with how the HRs are presented in the results section – it is a bit distracting to see the results presented so many different ways (e.g. mix of HRs inside parentheses or just placed at end of the sentence).

**Response:** One convention was adopted throughout the results section, “(HR=N.NN, CI: N.NN, N.NN)

**Discussion
Reviewer 2 Comment 9:** In general, I think the discussion needs more focus on the public health impact and potential actions/interventions from the results since you say in the abstract that these results are useful for overdose prevention targeting.

**Response:** We appreciate this recommendation and have responded by attempting to expand on potential actions/interventions relevant to SES variables in the discussion.

**Reviewer 2 Comment 10:** I think there are also some opportunities to discuss harm reduction (e.g. naloxone co-prescribing in the context of pain treatment) that are not mentioned.

**Response:** Line 313 to 314 states “*This effort may require clinician education to distinguish between opioid use disorder and physical dependence to opioids, [32] while maximizing harm reduction efforts (e.g. naloxone co-prescribing in the context of pain treatment).”*

**Reviewer 2 Comment 11:** The paragraph on health insurance is somewhat unclear - I’m not sure how the final two sentences connect to the results and the potential public health implications of this result aren’t clear. For example, should we increase insurance coverage and/or find ways to engage those without health insurance with harm reduction and addiction treatment services?
**Response:** Beginning on line 308, the paragraph has been revised to make clearer connections between expanded access to care and the role of providers.

*“In responses to the national opioid epidemic, individuals affected by addiction who are uninsured may be more able to access critical healthcare resources now than in the past, including mental health and substance abuse services. [30] As healthcare services become more accessible to people at-risk of opioid-related fatality, heightened attention has also been given to responsibilities of prescribers to distinguish between legitimate prescriptions for pain management and the prescription of opioids that could be diverted for non-medical purposes. [31]”*

**Reviewer 2 Comment 12:** One explanation for the higher overdose mortality among separated, divorced, or widowed people are that they may be more likely to use alone, conferring a higher fatal overdose risk.

**Response:** On line 255 this sentence was added,

*“Being in a marital relationship or other domestic partnership may limit time spent alone, and resulting social isolation that may predispose to fatal opioid overdose. [29]”*

**Reviewer 2 Comment 13:** How are the first and second parts from this sentence are connected: “The availability and lethality of fentanyl has contributed to an emerging pattern of post-incarceration overdose deaths, with a median time from release to death of more than 90 days [40].”

**Response:** On line 325-327, this sentence was revised to,

*“**These later onset post-incarceration deaths were not thought to be primarily due to reduced tolerance but a consequence of the lethality of (mostly illicit) fentanyl when eventually encountered. [52]”*
