# Supplementary material for: Socioeconomic risk factors for fatal opioid overdoses in the United States: Findings from the Mortality Disparities in American Communities Study (MDAC)
Source: PLoS One. 2020 Jan 17;15(1):e0227966. doi: 10.1371/journal.pone.0227966 (PMC6968850; doi:10.1371/journal.pone.0227966)
Supplement: Attachment — Submitted filename: PONE-D-19-16947 point by point responses December 2 2019.docx [file pone.0227966.s003.docx]

**Scientific Editor**:

**Comment 1**. As you revise your manuscript according to the minor revisions suggested by the two reviewers, I also encourage you to give some additional thought to the relationship between race, SES and fatal overdose.

**Response:** This essential comment was address in the (new) second paragraph of the discussion on lines 252-254. Please see detailed response to Reviewer 2, Comment 10.

**Comment 2**. As reviewer #2 has suggested, consider presenting the partially adjusted results in light of the complex relationships between SES and race.

**Response:** This excellent suggestion was adopted in the results on lines 194-196. Please see response to Reviewer 2, Comment 10 below.

**Reviewer #1:**

**Comment 1**. Abstract: Refrain from using stigmatizing language. Replace “disabled people” with “people with disabilities”

**Response:** Throughout the manuscript we have attempted to adopt this suggestion.

**Comment 2**. Introduction: The authors have done a nice job of summarizing the literature on the opioid overdose epidemic. An explanation of the gaps and limitations in the literature would round out this section so that the reader can understand the novelty of the current study.

**Response:** In the introduction, from line 71 to 78, we have included this explanation:

“Although data on SES attributes including education, income, and employment are available at the county [11] and census tract-level [12], the gold standard for analysis is use of individual-level data to examine effects of personal attributes. There is a paucity of individual-level data on prospective relationships between individual-level SES measures and risk of fatal opioid overdose, including for critical factors such as health insurance coverage, employment and marital status, and incarceration. [13]

National surveillance systems for opioid mortality typically do not capture detailed individual-level SES data. [1, 5, 6, 7, 8] Well-designed studies that include these data are often set in smaller geographic areas such as states, [10] and are not generalizable to the U.S. population.”

**Comment 3**. Results: Given the large sample size, I suggest reporting one decimal place for all percentages, both in the text and the tables.

**Response:** This recommendation was followed in both Table 1 and the results text.

**Comment 4**. In table 2, level of statistical significance would likely be of interest to readers. I would indicate using footnotes (e.g., * p <0.05, ** p<0.001 etc.)

**Response:** This recommendation was accepted in revision of Table 2.

**Comment 5**. Discussion: Another limitation is that medication and behavior therapy coverage was not measured. The authors may want to mention this fantastic consensus report that highlights national gaps in coverage <https://www.nap.edu/catalog/25310/medications-for-opioid-use-disorder-save-lives>

**Response:** The recommendation is used in new paragraph of the discussion, with the inclusion of reference 32, lines 259-275:

“People who were disabled had almost three times higher risk of death from opioid overdose than those without a disability, likely reflecting use of opioid analgesics to treat chronic pain. In 2016, CDC published guidelines to assist prescribers in weighing the benefits and risks of opioid therapy for chronic pain. [29] In 2019, the guidelines were evaluated by a consensus panel [30] and the CDC published a perspective [31] on measures to prevent misapplications of the guidelines that can cause harm. Another consensus report highlighted national gaps in evidence-based coverage that can save lives. [32] Examples include inflexible application of dosage and duration thresholds, abrupt tapering of opioid dosages, drug discontinuation, or dismissal of patients from care. Misapplication of the guidelines to other patient populations is another concern. This includes patients with pain at end-of-life, from cancer, acute surgical recovery, or sickle cell crises. Application of chronic pain dosage guidelines when prescribing opioid agonists to treat opioid use disorder can also cause harm. A need exists for empathetic chronic pain management such that non-opioid treatment is provided to the need for opioids, while taking into consideration the risks associated with each type of treatment. When patients agree to taper the dose of opioids, it is helpful for the pace to be individualized and gradual, to minimize withdrawal symptoms. [32] Further research on alternative chronic pain management strategies could point to interventions that lower opioid overdose mortality among patients at risk for opioid use disorder because of their medical comorbidities. [33]”

**Comment 6**. Minor comments, Abstract:

Replace “individual” with “individual-level” in the second sentence of the abstract.

**Response:** Lines 1-2 of the abstract were revised to incorporate this suggestion:

“Understanding relationships between individual-level demographic, socioeconomic status (SES) and U.S. opioid fatalities can inform national interventions in response to this crisis.”

**Comment 7**. In the results, “those with the highest level of educational attainment of high school” does this include college? This finding, as written, does not standalone.

**Response:** Table 1 and 2 now include the category **“**High School/GED only.” On line 177-179, this sentence was revised:

“People whose highest level of educational attainment was a High School diploma or GED only, with no college accounted for…”

On line 216 similar terminology is used:

“HRs among those with attainment of a High School diploma or GED only…”

**Reviewer #2:**

**Comment 1**. Throughout: use person-first language where you are able. For example, line 68: “the homeless, and recently released prisoners” could be changed to persons who have experienced homelessness, housing instability, or incarceration. Also line 285 “substance users” should be changed to persons who use substances

**Response:** The manuscript was revised to use person-first language throughout. On line 308 “substance users” was revised to “people who use substances.”

**Comment 2**. Line 24: “Opioid fatalities are a U.S. problem.” – I think the prior version’s topic sentence was stronger and you might remove this sentence in favor of expanding the second sentence’s justification for the analysis.

**Response:** The sentence beginning on line 24 was revised as suggested:

“Understanding relationships between individual-level demographic, socioeconomic status (SES) and U.S. opioid fatalities can inform national interventions in response to this crisis.”

**Comment 3**. Line 29: Likewise “HRs for fatal opioid overdose by SES in final model:” in the results section is not needed. Clearer to go straight to the results you interpret.

**Response:** The abstract was revised as recommended on line 30-31:

“In multivariable analyses, compared to…”

**Comment 4**. Line 41: “West North Central state”, do you mean “West, North, or Central”? Potentially missing a word or were these areas combined?

**Response:** The “West North Central” States are one Census district that includes Missouri, Kansas, Iowa, Nebraska, the Dakota, Minnesota, and Wisconsin. Due to abstract word limits, this terminology was retained.

**Comment 5**. Lines 45-46: Could strengthen the conclusions by suggesting how these findings from MDAC inform prevention, treatment, and rehabilitation (e.g. what communities or regions need more services?)

**Response:** Within constraints of abstract word limits the following revision was made on lines 45-46:

“ The findings may help to target prevention, treatment and rehabilitation efforts to vulnerable groups.”

**Comment 6**. Introduction lines 69-71: “Some risk factors such as unemployment and health insurance status are optimally studied using individual-level longitudinal data, with adjustment for other covariates. [11]” I do not get a lot of information from this sentence about what is known about the relationship of employment and health insurance coverage with opioid overdose. Could you instead highlight the main finding(s) of citation 11?

**Response:**  Lines 71-75 were revised to indicate that individual-level data is the gold standard for analyses of SES:

**“**Although data on SES attributes including education, income, and employment are available at the county [12] and census tract-level [13], the gold standard for analysis is use of individual-level data to examine effects of personal attributes. There is a paucity of individual-level data on prospective relationships between individual-level SES measures and risk of fatal opioid overdose, including for critical factors such as health insurance coverage, employment and marital status, and incarceration. [14]

**Comment 7**. Introduction, lines 77-78: “after multivariable adjustment” – It may be clearer to say you hypothesized these would remain independently associated with fatal opioid overdose even after adjustment for other socioeconomic and demographic indicators (or something similar). ‘Multivariable adjustment’ on its own leaves the reader unclear on what you are doing.

**Response:**  This comment was integrated with authors instruction for the introduction, to “conclude with a brief statement of the overall aim of the work and a comment about whether that aim was achieved.” Lines 82-83 were revised as follows:

“The MDAC database supported our aim to estimate hazard ratios for demographic, geospatial, and individual-level SES risk factors and fatal opioid overdose in the United States.”

**Comment 8**. Methods, lines 93-95: “Representativeness of the sample relative to the United States population was enhanced by applying weights to account for variable sampling.” – I don’t think you need this sentence and it makes the description of sampling weights less clear. The phrase ‘variable sampling’ is vague – unclear if variable is used as an adjective (as in varying) or noun (as in age, sex, etc. variables used for sampling).

**Response:**  When lines 94-101 were revised, addressing sample weights, this sentence was deleted:

“The sample frame for the ACS is derived from the Master Address File. Sampling is designed to approximate age, sex, race, Hispanic ethnicity, and state of residence distributions observed in the Census Bureau’s annual United States population estimates. Whites were oversampled by about 2% compared to the 2010 Census, and therefore observations for this group were assigned weights of slightly less than one. Other racial/ethnic groups were undersampled by one percent or less, given weights slightly above one. Overall weights were centered on one observation per respondent i.e., the weighted sample approximates the number of 2008 ACS responses rather than the U.S. population. Table cell counts were rounded to four significant digits to prevent disclosure of identity.”

**Comment 9**. Results, line 156: “weighting to the U.S. population” could be clearer if you say “weighting to the age, sex, etc…distribution of the U.S. population.” Otherwise wording could imply that the sample size is weighted to the size of the US population and someone reading quickly or without much background in survey data analysis might misunderstand that only 3800 opioid overdoses occurred in the US if not clarified that the exact numbers do not represent the US population size.

**Response:**  Lines 148-149 was revised as follows:

“After… weighting to age, sex, race, Hispanic ethnicity, and state of residence distributions of the U.S. population…”

**Comment 10.** The result in the final model that black participants were at lower risk of fatal overdose should be considered more thoroughly. The unadjusted and partially adjusted models suggest black race is a risk factor for fatal overdose and this association only becomes protective after adjusting for many SES related factors that disproportionately affect African American communities. A model considering race as a main exposure should not be adjusted for SES factors because race would predict some of those SES indicators but the SES factors would be mediators of relationships between race and opioid fatality (i.e., direction of causality is not SES factors predicting race which would be required for SES factors to confound relationship of race and overdose fatality). Thus the partial model may be more relevant here. I would suggest stating both the partial and full model results for race in your results and interpreting some of this in the discussion because it suggests that some attention to structural factors disproportionately affecting African Americans could decrease opioid overdose mortality among African Americans. Presenting only the full model results of a protective relationship suggests that African American communities are less affected by the opioid epidemic, which may not necessarily be the full story here.

**Response:**  In the results, on lines 194-196, the less adjusted model results are described:

“ In univariate and partially adjusted models Blacks had increased hazard rates (HR=1.37, CI: 1.16, 1.60) and (HR=1.44, CI: 1.22, 1.69), respectively, although those rates became lower than 1 after further adjusting for SES in the final model (HR=0.81, CI: 0.68, 0.96).”

Also, in the paragraph 2 of the discussion (lines 243-254) we address this important comment:.

“If SES partially mediated the effect of race in the final model, then interventions that impact SES, such as improving education, may have among their many advantages, the ability of help decrease opioid overdoses and associated racial disparities. [25-28]”

**Comment 11.** Line 248: Might consider making the statement “patients using opioid therapy for chronic pain should be carefully selected” a bit more specific and actionable by citing CDC opioid prescribing guidelines (Dowell et al CDC Guideline for Prescribing Opioids for Chronic Pain--United States, 2016). Could say something like: “physicians should weigh the benefits and risks of opioid therapy using national guidelines for opioid prescribing.” More recently these guidelines have been evaluated and you could also evaluate that literature in rewriting this sentence.

**Response:** Paragraph 3 of the discussion (lines 259-275) was expanded to include the CDC guidelines by Dowell et al (ref 29) and the Consensus panel report (ref 30) and CDC commentary (ref 31). A National Academies of Sciences, Engineering, and Medicine consensus report on opioid use disorder (ref 32) was also included at the recommendation of another reviewer of this manuscript:

“People who were disabled had almost three times higher risk of death from opioid overdose than those without a disability, likely reflecting use of opioid analgesics to treat chronic pain. In 2016, CDC published guidelines to assist prescribers in weighing the benefits and risks of opioid therapy for chronic pain. [29] In 2019, the guidelines were evaluated by a consensus panel [30] and the CDC published a perspective [31] on measures to prevent misapplications of the guidelines that can cause harm. Examples include inflexible application of dosage and duration thresholds, abrupt tapering of opioid dosages, drug discontinuation, or dismissal of patients from care. Misapplication of the guidelines to other patient populations is another concern. This includes patients with pain at end-of-life, from cancer, acute surgical recovery, sickle cell crises. Application of chronic pain dosage guidelines when prescribing opioid agonists to treat opioid use disorder can also cause harm. A consensus report highlights national gaps in evidence-based care for opioid use disorder that can save lives. [32] A need exists for empathetic chronic pain management such that non-opioid treatment is provided to the need for opioids, while taking into consideration the risks associated with each type of treatment. When patients agree to taper the dose of opioids, it is helpful for the pace to be individualized and gradual, to minimize withdrawal symptoms. [32] Further research on alternative chronic pain management strategies could point to interventions that lower opioid overdose mortality among patients at risk for opioid use disorder because of their medical comorbidities. [33]”
