# Supplementary material for: Socioeconomic risk factors for fatal opioid overdoses in the United States: Findings from the Mortality Disparities in American Communities Study (MDAC)
Source: PLoS One. 2020 Jan 17;15(1):e0227966. doi: 10.1371/journal.pone.0227966 (PMC6968850; doi:10.1371/journal.pone.0227966)
Supplement: Attachment — Submitted filename: Reviewer comments.docx [file pone.0227966.s001.docx]

This epidemiological study examines the relationship between multiple indicators of SES and fatal overdose in a nationally representative sample and fills an important gap in the overdose literature. The researchers present data drawn from the American Community Survey, which has been linked to fatal overdose data from the National Death Index. While this is an important topic with implications for targeted overdose prevention and response programs, grounding the research in the large body of existing literature on fatal overdose would strengthen the paper. There are also several methodological issues as well as questions around generalizability that need to be addressed. With minor revisions, this paper could be a timely contribution to the overdose literature.

Abstract

The results section of the abstract should be revised; there appears to be incomplete reporting e.g., urban group had higher risk than the rural group but was not reported, “HRs near two” etc and the wording is convoluted at times. The abstract refers to “final models” when the results only report one final model. I suggest rewriting the results section of the abstract to be more concise and more informative.

Introduction

Page 3: Nationwide, fatal opioid overdoses have disproportionately [2] but not exclusively [3] affected men, and particularly adolescent, young and middle-aged adult white men. -Clarify whether this is referring to fatal opioid overdose prevalence or rates over time and the historical period of relevance.

It would be worth mentioning the reason why overdose has been conceptualized as a death of despair to strengthen the rationale for focusing on SES, and to cite the original paper that developed the theory. The cited reference does not use the phrase and so the intended connection is unclear.

Given that this is a national study, more could be provided in the introduction about the relationship between overdose rates and geography to better contextualize the findings (e.g., urban vs. rural settings), including whether overdoses or low SES are known to be concentrated in certain locales.

Much has been written about the current opioid epidemic. A description of the established risk factors of fatal overdose or at minimum including citations of published reviews (e.g., Dasgupta et al., AJPH; Martins et al., AJPH) would nicely set up the subsequent sections and lay the foundation for the known confounders (measured and unmeasured) that should be considered.

Methods:

Page 4: (12.8%) ACS respondents who were missing information necessary to meet the NCHS minimum criteria required for matching – did the authors conduct secondary analyses to understand the degree of bias due to this missingness e.g., if this group differed on socio-demographics?

Page 5: The Census Bureau definition of rural [14] excluded metropolitan and micropolitan areas, incorporated census-designated places with populations of 2500 or more. All residential locations were classified as rural or urban. – This needs to be unpacked; it is an important factor with implications for targeting resources. It appears that rural vs. non-rural would be a more accurate description based on the current text. Could the authors instead use a more granular classification scheme?

Page 6: Analyses that accounted for multistage sampling in the ACS were conducted using the PHREG procedure – clarify the number of levels included in the final model. Did this method account for correlations between individuals surveyed from the same area of residence (e.g., census division)?

Page 6: incarceration at time of survey – clarify here and in the table whether this is referring to the setting in which the survey was conducted or if the individual had a history of incarceration.

Page 6: Were any confounders considered for inclusion in the regression analysis? Access to naloxone is an example. If not, this should be stated in the limitations.

Results:

Page 8: Many variables as modeled contain extremely small cell sizes (e.g., n=36 among ages 80+; n=29 among Asian Pacific Islanders; n=43 American Indian Alaskan Native etc), which calls into question the validity of these results. Where appropriate, I would suggest re-categorization/collapsing those categories into another category to generate larger cell sizes and/or replacing reference groups containing small cells with new reference groups with larger samples; this will affect the precision of the Cox regression models.

Table 2: Clarify whether the presented data are from the Final adjusted model or the Partially adjusted model

Discussion

Page 22: This nationally representative descriptive study – given that the paper includes data analysis, it is observational rather than descriptive.

Page 22: Given that the term “opioid” is often used synonymously with prescription opioid, I suggest highlighting in the opening paragraph that the analysis also included deaths involving non-medical opioids including heroin and fentanyl.

Page 22: What do the authors mean by “rehabilitate those at-risk for opioid overdose?” Replacing this with less stigmatizing language is recommended.

Page 22: were any subpopulations undersampled? E.g., Asian Pacific Islanders; American Indian Alaskan Native Americans. How valid are the findings around race/ethnicity?

Page 23: How does the poverty measure used account for regional differences in cost of living?

Page 24: Compared to people who owned a house with a mortgage, renters or owners without a mortgage were at risk of fatal opioid overdose during the study period – what was the hypothesis? Was this a surprising finding? I would suggest explaining this finding further.

Page 24: The availability and lethality of fentanyl – I suggest clarifying that it is mostly illicitly manufactured fentanyl.

Page 24: Increased access to opioid agonist therapy for treating opioid use disorder may have merit, but currently adherence rates are low – it is unclear what is meant by “low” and what the possible underlying reasons may be. This statement does not add to the discussion and could be removed.

Limitations: How stable are the SES indicators over time? It would be helpful if the authors could explain which SES variables are most susceptible to temporal change and which remain relatively stable over time.

Limitations: Homelessness is highly prevalent among people who misuse opioids, and many of those who overdose are unstably housed. Same with individuals who are incarcerated at the time of the study. How does the ACS account for homeless and incarcerated individuals? If this is a limitation of the study, please state.

Minor comments: typographic errors should be fixed throughout.
